# Circ_0021350 plays an oncogene role by regulating miR-1207-3p/PIK3R3 in glioblastoma

**DOI:** 10.1186/s12885-023-11263-w

**Published:** 2023-08-29

**Authors:** Cheng Tan, Jun Wei, Zhaohui Li, Nan Tian, Zhengming Wang, Guan Wang, Liang Han, Yu Tian

**Affiliations:** 1 Department of Emergency, China-Japan Union Hospital of Jilin University, Changchun, Jilin China; 2 Clinical Research Laboratory Phase I, China-Japan Union Hospital of Jilin University, Changchun, Jilin China; 3 Department of Neurosurgery, China-Japan Union Hospital of Jilin University, Changchun, Jilin China; 4 College of Life Science, Zhejiang Chinese Medical University, Hangzhou, Zhejiang China; 5 Department of Pathology, China-Japan Union Hospital of Jilin University, Changchun, Jilin China

**Keywords:** CircRNAs, Glioblastoma, miR-1207-3p, PIK3R3, RNA-seq

## Abstract

**Background:**

Glioblastoma (GBM) is the most malignant glioma, with poor survival rates and prognosis. Several studies have reported the abnormal expression of circular RNAs (circRNAs) and their functions in the malignant biological behavior of GBM. However, such research is still in the preliminary stages, and further study is needed to confirm the therapeutic potential of circRNAs in GBM.

**Methods:**

RNA-seq was performed using four tumor tissues from patients with GBM and their adjacent non-tumor brain tissues to screen differentially expressed circRNAs. Fluorescence in situ hybridization assay was used to examine the location of circ_0021350 in glioma cells. In addition, a series of biological function assays were employed to verify the oncogenic role of circ_0021350 in GBM. Quantitative reverse transcription PCR was used to examine circular, micro- (miRNA), and messenger RNA (mRNA) levels. Furthermore, dual-luciferase reporter, RNA pull-down, and RNA binding protein immunoprecipitation assays were applied to verify the interaction between circ_0021350 and its downstream effectors.

**Results:**

Circ_0021350 was significantly elevated in GBM tissues and glioma cells. Overexpression of circ_0021350 promoted glioma cell proliferation and metastatic ability; silencing of circ_0021350 had the opposite effect. Mechanistic analysis revealed that circ_0021350 sponged miR-1207-3p to regulate PIK3R3, whose overexpression reversed the reduction in the malignant biological behavior of glioma cells caused by silencing circ_0021350.

**Conclusion:**

Our findings suggest that circ_0021350 is an oncogenic circRNA in GBM, and the circ_0021350/miR-1207-3p/PIK3R3 axis may serve as a potential therapeutic target in GBM treatment.

**Supplementary Information:**

The online version contains supplementary material available at 10.1186/s12885-023-11263-w.

## Introduction

In recent years, the ENCODE project has made the non-protein-coding region of the genome a research hotspot in life sciences, including cancer biology. Accumulating evidence suggests that aberrant non-coding RNAs (ncRNAs) expression is involved in cancer initiation, progression, and resistance to therapy at the transcriptional, post-transcriptional, and epigenetic levels [1, 2]. Novel types of ncRNAs, also known as circRNAs, have become a new class of potential tumor biomarkers owing to their stable structure, rich content, and tissue-specific expression [3–5]. Unlike linear RNA, circRNAs exist as covalently closed loops without a 3′ polyadenylate tail or 5′ cap and are synthesized from the reverse splicing of special pre-mRNAs in a non-classical way [4]. Research has shown that circRNAs function mainly as miRNA sponges, regulating RNA-binding protein (RBPs) activity or translation to proteins [6]. Further exploration of their functions will provide great benefits for early diagnosis and prognostic prediction of cancers.

Gliomas are characterized by high mortality and recurrence rates and account for more than 70% of all primary brain tumors [7]. Although surgery combined with radio/chemotherapy has remarkably improved quality of life, the prognosis of patients with glioma remains poor [8]. For glioblastoma (GBM), a World Health Organization (WHO) grade IV glioma, the median survival period is still short (12 ~ 15 months) with a 5-year survival rate of only 4.7% [9]. The reasons for poor prognosis are the rapid growth of the tumor tissue, extensive infiltration into the adjacent brain tissue, further induction of pseudonecrosis, abundant angiogenesis, and the absence of a therapeutic target [10]. Therefore, more effective strategies should be explored to improve the diagnosis and prognosis of GBM. Kadener et al. [11] reported high enrichment and specificity of circRNAs in the central nervous system, indicating their association with brain disorders, including GBM. Several research groups have reported abnormal expression of circRNAs and their functions in malignant glioma behavior [12–14]. However, current research on circRNAs is still in the preliminary stages, and further research is needed to confirm whether they can be used as therapeutic targets or diagnostic and prognostic markers for GBM.

To screen for differentially expressed circRNAs in GBM, we performed RNA-seq using four tumor tissues and adjacent non-tumor brain tissues from patients with GBM and focused on circ_0021350, which is upregulated in GBM. We found that circ_0021350 promoted the proliferation of the glioma cells both in vitro and in vivo. Using bioinformatics prediction, we identified the binding sites for miR-1207-3p in circ_0021350, and subsequent experiments further verified their regulatory relationship. We also confirmed that PIK3R3 is a target of miR-1207-3p and that circ_0021350 indirectly regulates its expression. Rescue experiments showed that overexpression of PIK3R3 reversed the reduction in malignant biological behavior caused by circ_0021350 knockdown. Mechanistically, circ_0021350 participates in GBM via the miR-1207-3p–PIK3R3 axis.

## Materials and methods

### Tissues specimens and cell lines

Four GBM and adjacent non-tumor brain tissue samples were obtained from patients who underwent surgery at the China-Japan Union Hospital of Jilin University (Changchun, China). GBM was diagnosed according to the 2021 WHO classification of CNS [15]. The study protocol was approved by the Ethics Committee of the China-Japan Union Hospital of Jilin University (Changchun, China), and all methods were performed in accordance with the relevant guidelines and regulations. Human glioma cell lines A172, U251, U87, and HA1800 normal astrocytes were obtained from Tian’s laboratory at Zhejiang Chinese Medical University. The cell lines were grown in DMEM plus 10% fetal bovine serum (Gibco, USA) at 37 °C supplied with 5% CO_2_.

Sequencing Library Preparation and circRNA Sequencing.

Total RNA was isolated from the specimens using the RNAiso Plus reagent (TaKaRa, Japan) in accordance to the manufacturer’s protocol. A NanoDrop ND-1000 spectrophotometer (Agilent, Santa Clara, CA, USA) was used to quantify the RNA. Total RNA from each sample was used to prepare the circRNA sequencing library via the following steps: (1) 2 µg total RNAs was enriched with oligo dT; (2) RNA libraries were constructed by using pretreated RNAs with KAPA Stranded RNA-Seq Library Prep Kit (Illumina, USA) following the manufacturer’s instructions; (3) the libraries were controlled qualitatively and quantitatively by using the Agilent BioAnalyzer 2100 system (Agilent Technologies, Inc., USA), denatured as single-stranded DNA molecules, captured on Illumina flow cells, amplified in situ as clusters, and finally, sequenced for 150 cycles on Illumina HiSeq4000 Sequencer according to the manufacturer’s instructions.

### Expression profile analysis of circRNAs

After image and base recognition, raw sequencing reads were obtained using an Illumina HiSeq Sequencer. For circRNA detection, raw reads and the human reference genome were compared using STAR software. The Edger software package was used for the differential expression of circRNAs. CircRNAs with fold changes ≥ 1.5, and *P*-values ≤ 0.05 were identified as significantly differentially expressed.

### RNase R treatment and PCR analysis

Total RNAs and genomic DNAs were isolated using TRIzol reagent (Ambion, USA) and a Universal Genomic DNA Kit (Cw Biotech, China), respectively. Quantitative Real-time PCR (qRT-PCR) was performed using the UltraSYBR One Step RT-qPCR Kit, and PCR was done using GoldStar Best DNA Polymerase (cwbiotech, China). The differences of circRNA and miRNA were normalized to the levels of β-actin or U6. Details of the primers used are listed in Table S1. For the RNase R treatment, 3 U/mg RNase R (KeyGENBioTech, China) was added to the reaction mixture and incubated at 37 °C for 15 min. The treated RNA was then transcribed into cDNA, and the expression of circ_0021350 and linear SOX6 mRNA was detected using qRT-PCR.

### Fluorescence in situ hybridization (FISH)

A172 and U251 cells (5 × 10^5^cells/well) were seeded in a 6-well plate and washed twice with PBS until they reached 80% confluence. To fix the cells, 4% paraformaldehyde (Biosharp, China) was added to the wells for 10 min, and then Cy3-labeled circ_0021350 and 18s probes were added to the cells separately at 37 °C overnight. DAPI was used to stain the nuclei, and fluorescence images were captured using a fluorescent microscope (ECLIPSE Ti-S, Nikon, Japan). All the probes were purchased from RIBoBio (Guangzhou, China).

### Cell transfection

Lentiviral vectors for the overexpression of circ_0021350 (pLVX-circ_0021350), PIK3R3 (pLVX-PIK3R3), and shRNAs of circ_0021350 were synthesized by Miaolingbio (Wuhan, China). Lipofectamine 3000 (Invitrogen, USA) was used for plasmid transfection according to the manufacturer’s instructions. The cells transfected with the lentiviral vector were screened with 2.0 µg/mL puromycin for six weeks. Circ_0021350 siRNAs, siRNA negative control (siRNA-NC), miR-1207-3p mimics, inhibitors, mimic negative control (mimic-NC), and inhibitor negative control (inhibitor-NC) were synthesized by Biomics Biotechnologies (Beijing, China) and transfected using HieffTrans reagent (Yeasen Biotechnology, China).

### Cell proliferation analysis

CCK-8 and EdU assays were used to assess the proliferative ability of the glioma cells. For the CCK-8 assay, the cells from each group were seeded into 96 well plates at a density of 3–5 × 10^3^ cells/well, and 10 µL of CCK-8 solution (Keygen biotech, China) was added into each well, which were incubated at 37 °C for 2 h. The OD_450_ was detected using a microplate reader RT-6000 (RAYTO, China). For the EdU assay, the cells from each group were seeded into 96 well plates at a density of 5 × 10^3^cells/well. When the cells adhered, 10 µM EdU reagent (Beyotime, China) was added into the wells at 37 °C for 2 h and then fixed with formaldehyde for 30 min. After staining with Hoechst33342, fluorescence images were captured using a fluorescence microscope (ECLIPSE Ti-S, Nikon, Japan).

### Colony formation assay

The cells in each group were seeded into 6-wells plates at a density of 500 cells/well and cultured at 37 °C for two weeks. After staining with 0.5% crystal violet, colonies were photographed, and the number of colonies was counted manually.

### Migration and invasion assays

A wound-healing assay was used to detect cell migration. The cells were seeded in 6-well plates at a density of 2 × 10^5^ cells/well and cultured in a serum-free medium. After incubating to confluence, the cells were scratched using a 200 µL pipette tip. Then, the plates were washed twice with PBS, and the area of the cell-free scratch was photographed at 0 and 24 h. A transwell invasion assay was used to detect cell invasion ability. The upper chamber of the Transwell insert (Corning, USA) was coated with Matrigel (Corning, USA), and the cells (3 × 10^4^/well) were seeded into the upper chamber. The upper chamber was incubated without FBS, and DMEM, supplemented with 10% FBS, was added to the lower chamber. Twenty-four hours later, the cells were fixed with 4% paraformaldehyde for 30 min and stained with 0.1% crystal violet. Non-invading cells in the upper chamber were carefully removed using a cotton swab.

### Luciferase reporter assays

Wild-type (wt) and mutant (mut) binding sequences of circ_0021350 or PIK3R3 3ʹUTR to miR-1207-3p were cloned into the pmirGLO vector (Promega, Madison, WI, USA). HEK-293T cells were seeded in 24-well plates at a density of 2 × 10^5^cells/well and transfected with the corresponding plasmids and miR-1207-3p mimics/mimics-NC. Luciferase activity was measured following the manufacturer’s instructions using a dual-luciferase reporter assay kit (Beyotime, China) and detected using a Centro LB960 XS3 (Berthold, Germany).

### RNA pulldown assay

Biotinylated circ _0021350 and NC probes were synthesized by Biomics Biotechnology (Beijing, China). A172 and U251 cells were lysed and subjected to ultrasonication and then incubated with probes overnight at 37 °C. Next, antibiotic-streptomycin magnetic beads (Invitrogen, USA) were added to the reaction mixture for another 4 h. After washing the beads, the pull-down complexes were extracted with TRIzol and further analyzed using qRT-PCR. The probe sequences are listed in Table S1.

### Western blot analysis

The cells in each group were lysed using RIPA buffer (Beyotime, China), as described previously [16]. Briefly, the proteins were separated using SDS-PAGE and electrophoretically transferred to the PVDF membranes (Invitrogen, USA). After the membranes were blocked in 1% BSA for 2 h, the primary antibodies was added and incubated overnight at 4 °C. Subsequently, the membranes were incubated with horseradish peroxidase-conjugated secondary antibodies for another 2 h at room temperature. The anti-PIK3R3 (27035-1-AP), anti-PCNA(10205-2-AP), anti-β-actin (81115-1-RR) antibodies, and goat anti-rabbit secondary antibody (SA00001-2) were purchased from proteintech. The intensity of the protein signals was detected using chemiluminescence (Aplegen Omega Lum W, USA). To avoid the influence between two adjacent bands above and below in same gel during exposure, we cut the blot membranes prior to hybridisation with antibodies.

### RNA binding protein immunoprecipitation (RIP) assay

The glioma cells were lysed using Lysis Buffer (Millipore, USA). After centrifugation, the cell extracts were incubated with magnetic beads bound to anti-Ago or anti-IgG antibodies (Millipore, USA). The beads were then incubated with protease K lysate at 55 °C for 30 min after washing with wash buffer. Finally, precipitated total RNA was extracted and purified. qRT-PCR was performed to detect the circ_0021350, miR-1207-3p, and PIK3R3 levels.

### In vivo tumor growth assays

BALB/c nude mice (male, 4 ~ 6 week) were purchased from Shanghai Slack Laboratory Animal Co. Ltd and raised in Zhejiang Chinese Medical University Laboratory Animal Research Center (Hangzhou, China). All methods were carried out in accordance with relevant guidelines and regulations. U251 cells (1 × 10^7^) from the control, Lv-circ_0021350-shRNAs transfected group, Lv-circ_0021350-shRNAs + miR-1207-3p antagomir co-transfected, and Lv-circ_0021350-shRNAs + pLVX-PIK3R3 co-transfected groups were subcutaneously injected into the nude mice. The tumors were measured every seven days after tumor formation, isolated, and weighed after sacrificing the mice. The animals were sacrificed by cervical dislocation under deep CO2 anesthesia. Tumor volume was calculated using the following formula: V = W^2^ × L × 0.5. The animal studies were approved by the Laboratory Animal Management and Ethics Committee of Zhejiang Chinese Medical University. Subsequently, the tumor tissues were fixed and analyzed using immunohistochemistry.

### Statistical analysis

Statistical data were analyzed using GraphPad Prism 8.0. Each experiment was repeated at least three times, and the data were shown as mean ± SD. Kaplan-Meier survival analysis was used to estimate survival distributions, and the log-rank test was used to assess the statistical significance between groups, using the median value as the cutoff. The Pearson’s correlation coefficient was used to determine the association between two quantitative variables. Comparisons between groups were analyzed using the Student’s *t*-tests. *P* values < 0.05 were considered statistically significant.

## Results

### Differential expression of circRNAs in tumor tissues from patients with GBM

**Fig. 1:**
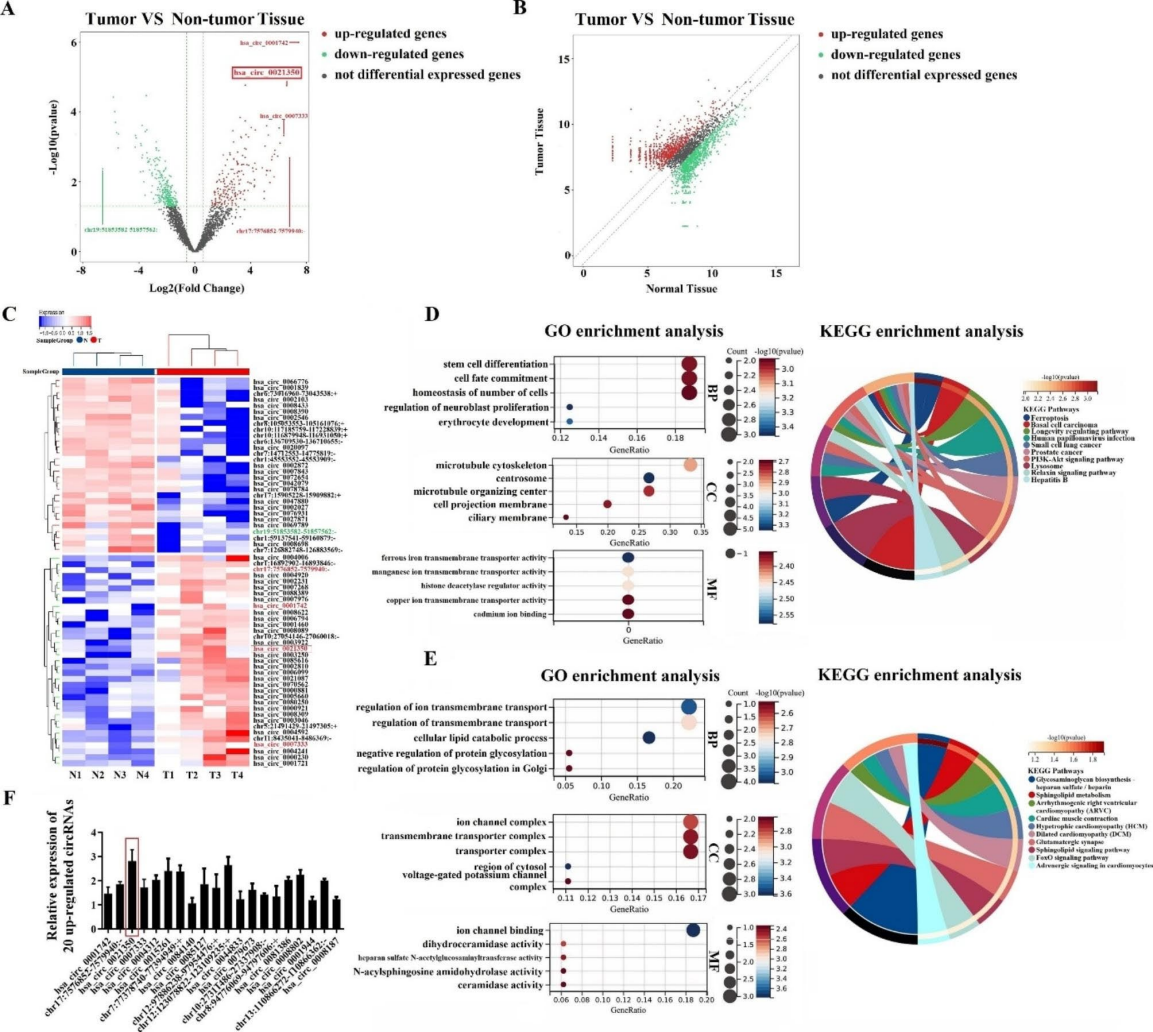
Identification of circRNAs by RNA-seq analyses in GBM samples. The volcano plot (**A**), the scatter plot (**B**), and hierarchical clustering analysis (**C**) showed the expression profiling of circRNAs between GBM and non-tumor tissues, the top 5 differentially expressed circRNAs are highlighted. GO and KEGG pathway enrichment analysis of the linear mRNAs of 20 mostly upregulated (**D**) and 20 mostly downregulated (**E**) circRNAs. (**F**) The expression levels of 20 mostly up-regulated circRNAs in GBM and non-tumor tissues detected by qRT-PCR.

To investigate the circRNA expression profiles in GBM tissues, we analyzed four GBM tissue samples and their adjacent non-tumorous brain samples. As shown in Fig. 1A, a total of 463 differentially expressed circRNAs (fold changes ≥ 1.5 and *P*-values ≤ 0.05) were identified in the patients with GBM using a Volcano Plot, including 197 upregulated and 266 downregulated circRNAs. Scatter plot analysis revealed variations in circRNA expression between the two groups (Fig. 1B), and hierarchical clustering analysis is shown in Fig. 1C. Next, GO and KEGG pathway analyses of 20 mostly upregulated and 20 downregulated circRNAs were conducted. GO analysis showed that the corresponding linear mRNAs of the 20 most upregulated genes favored stem cell differentiation, cell fate commitment, and regulation of neuroblast proliferation in BP, centrosomes and microtubules in CC, and transmembrane transporter activity and histone deacetylase regulator activity in MF. KEGG pathway analysis indicated that ferroptosis, basal cell carcinoma, the PI3K-Akt signaling pathway, and pathways in cancer were enriched (Fig. 1D). Regarding the corresponding linear mRNAs of the 20 downregulated circRNAs, BP was mainly enriched in cellular lipid catabolic processes, transmembrane transport regulation, and protein glycosylation; CC was mainly enriched in the cytosol, ion channel complex, and transporter complex; and MF was mainly enriched in ion channel binding and dihydroceramidase activity. In addition, KEGG analysis revealed that these genes were mainly enriched in glycosaminoglycan biosynthesis, sphingolipid metabolism, glutamatergicsynapses, and other cancer-related signal transduction pathways (Fig. 1E). Since functional analysis provided significant evidence to interpret the functions of the 20 upregulated circRNAs in the oncogenesis and growth of tumors, we chose to detect the expression levels of the 20 most upregulated circRNAs in the GBM and non-tumor samples using qRT-PCR. As shown in Fig. 1F, qRT-PCR results were consistent with the RNA-seq data. Among them, we found that circ_0021350 expression was markedly upregulated in the glioma tissues compared to the controls. Therefore, in this study, we focused on the expression and role of circ_0021350 in GBM progression.

### Circ_0021350 is upregulated in the glioma tissues and cell lines and both localized in the cell cytoplasm and nucleus

**Fig. 2:**
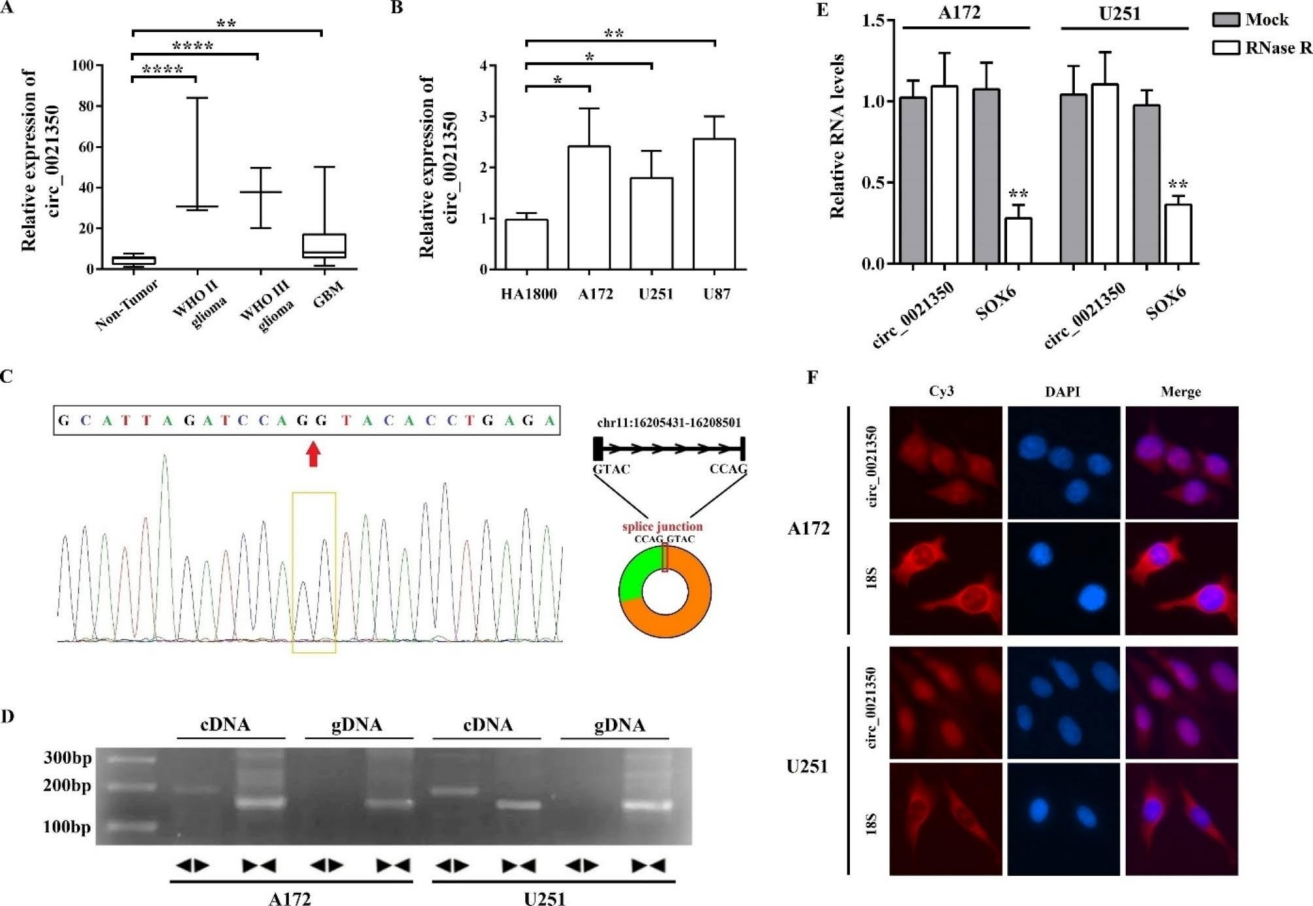
Identification and characterization of circ_0021350. (**A**) Relative expression of circ_0021350 in glioma and non-tumor tissues from data of Song et al. (**B**) Compare the expression of circ_0021350 in glioma cell lines and human astrocyte cells. (**C**) Sequencing after RT-PCR was used to show the splicing junction of circ_0021350. (**D**) PCR with divergent or convergent primers indicating the existence of circ_0021350. Here, we used cropped gel image and the original image was in additional files. (**F**) RNA-FISH indicated the location of circ_0021350. 18 S was used as cytoplasm control. ^*^P < 0.05, ^**^P < 0.01, ^***^P < 0.001, ^****^P < 0.0001

An independent dataset reported by Song et al. [17] was used to verify circ_0021350 expression in glioma tissues. This dataset showed increased circ_0021350 expression in the gliomas and decreased expression in normal brain tissues (Fig. 2A); this is consistent with our RNA-seq and qRT-PCR results. Next, we detected the expression pattern of circ_0021350 in glioma cell lines and found that it was upregulated in the A172, U251, and U87 cells compared to the normal human astrocyte HA1800 cells (Fig. 2B).

The genomic structure indicated that circ_0021350 consisted of two exons (242 bp) of SOX6 (Fig. 2C). The circular product was amplified by RT-PCR using divergent primers and confirmed by sequencing. As shown in Fig. 2D, circ_0021350 was amplified by divergent primers in cDNA, but not in gDNA, whereas linear SOX6 was amplified by convergent primers in both cDNA and gDNA. RNase R was used to pretreat the RNAs, and the results indicated that circ_0021350 was resistant to RNase R (Fig. 2E). Subsequently, FISH was performed to determine the subcellular localization of circ_0021350. The results indicated that circ_0021350 was localized in both the cytoplasm and the nucleus (Fig. 2F).

### Effects of circ_0021350 on cell cellular proliferation and metastasis

**Fig. 3:**
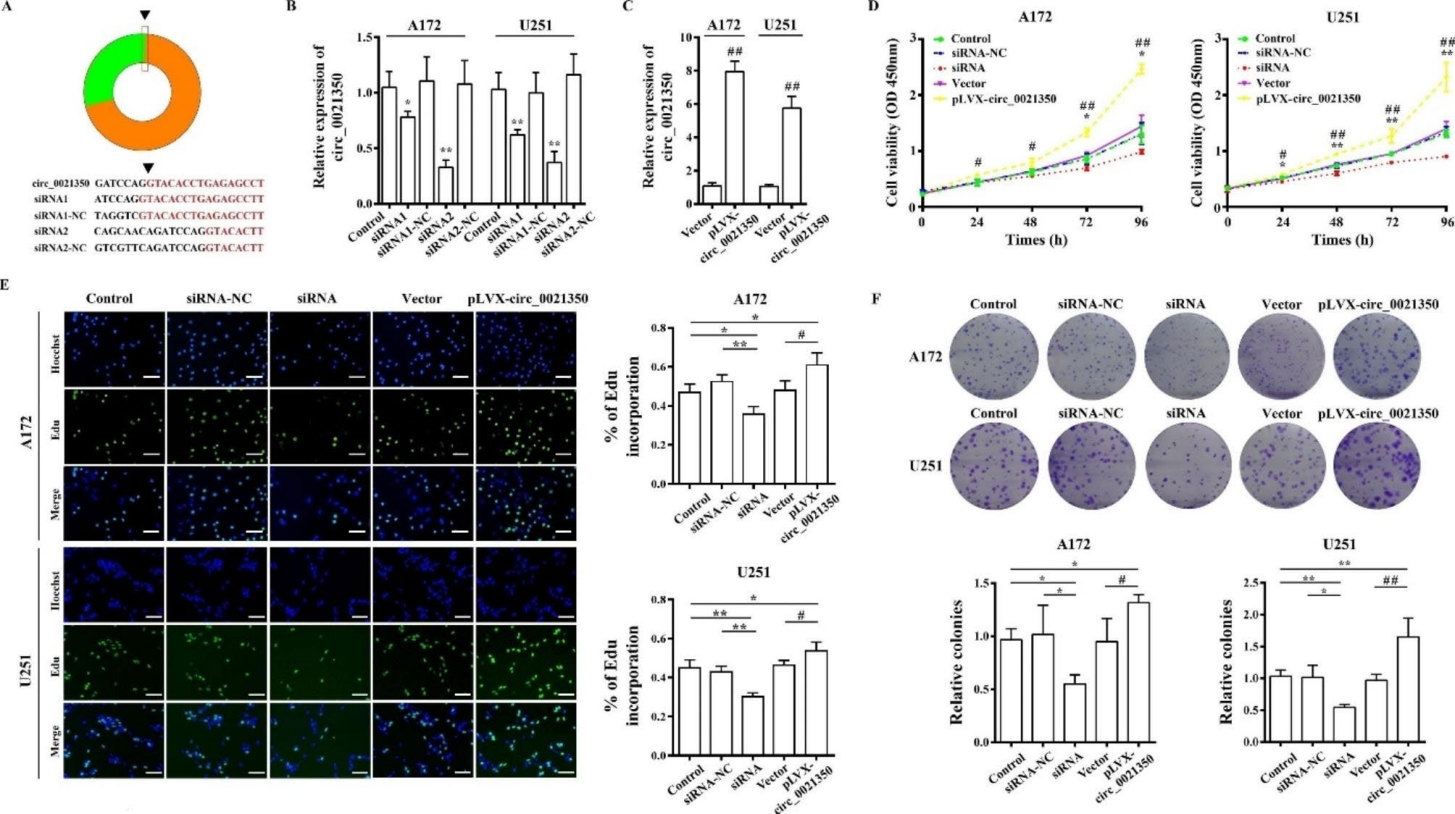
Effects of circ_0021350 on the proliferation and clonogenic capacity of glioma cells. (**A**) The siRNAs targeted the junction site of circ_0021350. (**B**) qRT-PCR results showed that siRNA-2 significantly reduced the expression levels of circ_0021350 and were used in followed experiments. (**C**) pLVX-circ_0021350 transfection increased the expression levels of circ_0021350 in glioma cells. (**D-E**) The proliferation rate of each group was examined by CCK-8 and EdU assays. Scale bars, 100 μm. (**F**) The clonogenic capacity of each group was examined by colony formation assay. ^*^P < 0.05, ^**^P < 0.01 compared to the siRNA group; ^#^*P* < 0.05, ^##^*P* < 0.01 compared to the pLVX-circ_0021350 group

**Fig. 4:**
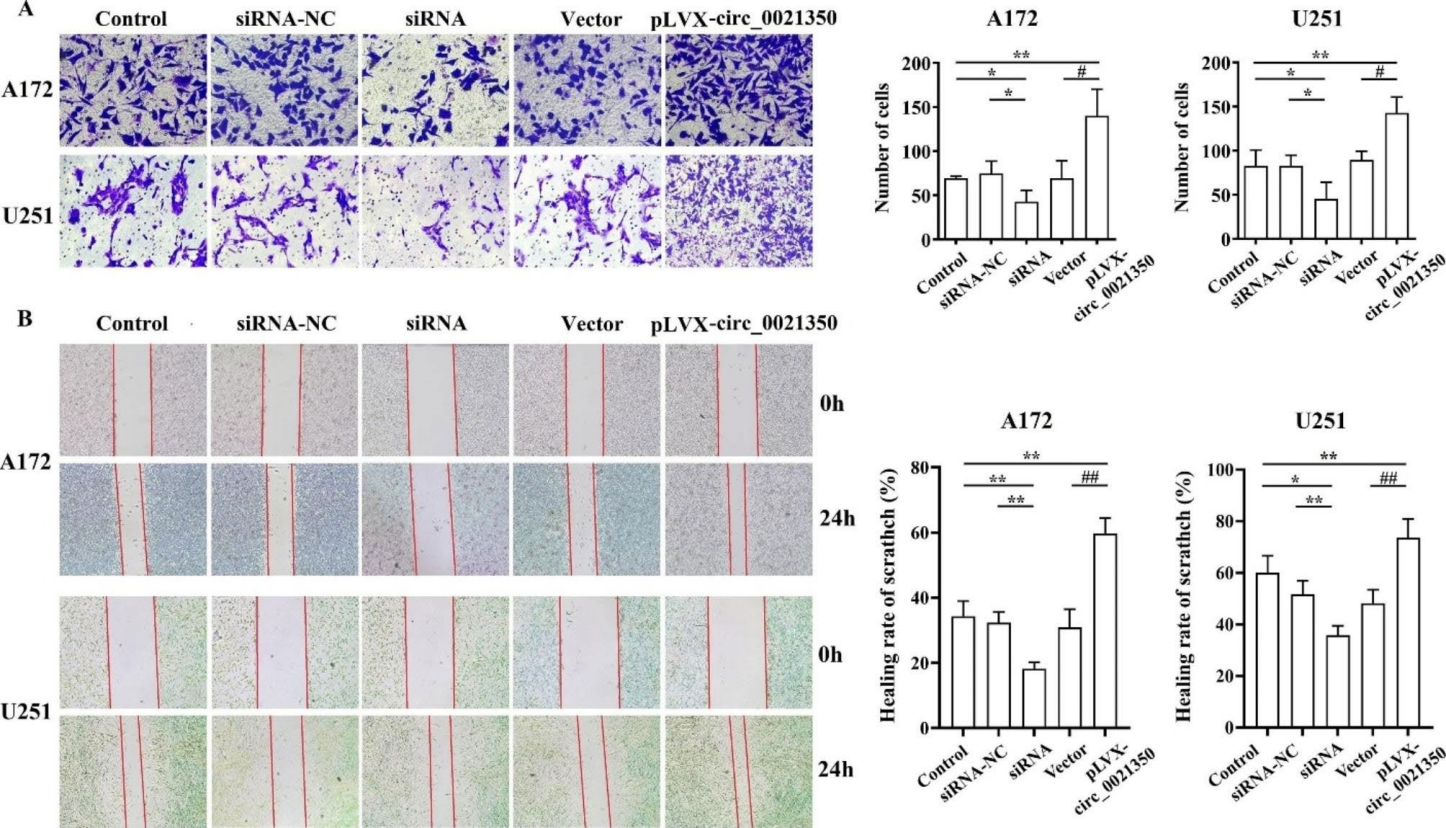
Effects of circ_0021350 on the metastasis ability of glioma cells. (**A**) The results of invasion ability of A172 and U251 cells in each group. (**B**) The results of migration ability of A172 and U251 cells in each group. ^*^P < 0.05, ^**^P < 0.01 compared to the siRNA group; ^#^*P* < 0.05, ^##^*P* < 0.01 compared to the pLVX-circ_0021350 group

To further study the biological function of circ_0021350, we knocked down the circ_0021350 using siRNAs and overexpressed circ_0021350 using the pLVX-circ_0021350 vector. CCK-8, colony formation, and EdU assays were performed. As shown in Fig. 3A-B, circ_0021350 expression levels were altered in A172 and U251 cells after transfection with siRNA-1 and siRNA-2, respectively. SiRNA-2 was selected for subsequent experiments because of its strong inhibitory effects. Moreover, circ_0021350 levels were markedly increased after transfection with pLVX-circ_0021350 (Fig. 3C). In the CCK-8 and EdU assays, we found that siRNA-2 transfection reduced the proliferative ability of the glioma cells compared to the control and siRNA-2 NC groups, while pLVX-circ_0021350 transfection increased the proliferative ability when compared to the control and empty vector groups (Fig. 3D, E). Furthermore, circ_0021350 knockdown notably reduced colony formation of A172 and U251 cells, whereas circ_0021350 overexpression increased the number of colonies (Fig. 3F). Transwell invasion assays demonstrated that circ_0021350 knockdown impaired the invasion ability, while circ_0021350 overexpression promoted this ability (Fig. 4A). In addition, wound-healing assays showed that circ_0021350 silencing markedly suppressed migration ability, whereas circ_0021350 overexpression promoted it (Fig. 4B).

### Circ_0021350 binds mir-1207-3p in glioma cells

**Fig. 5:**
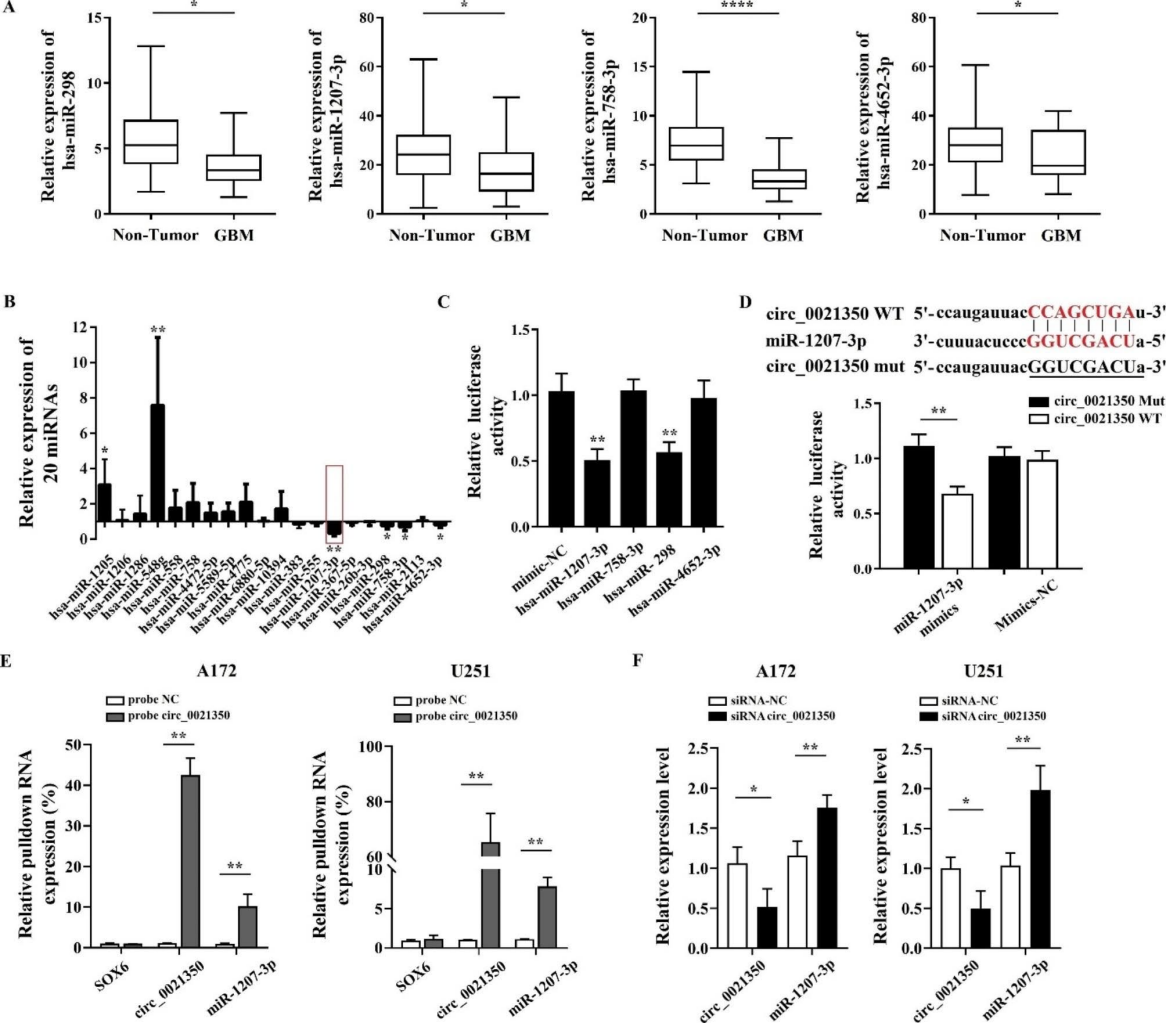
Circ_0021350 acts as a sponge for miR-1207-3p. (**A**) Differential expression of predicated miRNAs in glioma and non-tumor tissues by GEO database GSE145510. (**B**) The expression levels of 20 predicated miRNAs in GBM and non-tumor tissues detected by qRT-PCR. (**C**) Pre-screen miRNAs that could bind to circ_0021350 using luciferase reporter assay. (**D**) The binding between circ_0021350 and miR-1207-3p was verified by luciferase reporter assay. (**E**) miR-1207-3p was enriched in circ_0021350 probes but not in NC probes by RNA pull-down assay. (**F**) Relative expression of circ_0021350 and miR-1207-3p in glioma cells transfected with circ_0021350 siRNA or siRNA-NC. ^*^P < 0.05, ^**^P < 0.01,^***^P < 0.001, ^****^P < 0.0001

The most well-investigated mode of circRNA action is the miRNA sponging ability, which regulates downstream gene expression [18]. Therefore, we screened for miRNAs potentially associated with circ_0021350 expression. Using miRDB and circinteractome software, 20 miRNAs were predicted as possible targets of circ_0021350. Next, we detected the expression of these 20 miRNAs in the glioma tissues using the GEO database (GSE145510) and found that hsa-miR-1207-3p, hsa-miR-298, hsa-miR-758-3p, and hsa-miR-4652-3p were downregulated in GBM tissues compared to the normal tissues (Fig. 5A). Subsequent qRT-PCR confirmed the lower expression of hsa-miR-1207-3p, hsa-miR-758-3p, hsa-miR-298, and hsa-miR-4652-3p in the GBM tissues (Fig. 5B). Next, we inserted a circ_0021350 fragment into pmirGLO luciferase reporter vectors and co-transfected miRNA mimics into 293T cells to identify the actual binding between the circ_0021350 and miRNAs. Compared to the mimic-NC-transfected group, hsa-miR-1207-3p and hsa-miR-298 showed significantly decreased luciferase activity (Fig. 5C). Finally, we selected hsa-miR-1207-3p with the largest downregulated miRNAs, for further analysis. To further verify the hsa-miR-1207-3p binding sites in circ_0021350, WT and mut pmirGLO luciferase reporter vectors were co-transfected into 293Tcells with miR-1207-3p mimics or mimic-NC. As shown in Fig. 5D, miR-1207-3p mimics notably reduced luciferase activity in the cells transfected with WT vectors but did not affect the cells transfected with the mut vector. Subsequently, an RNA pull-down assay was performed to confirm the direct binding between the circ_0021350 and miR-1207-3p. As shown in Fig. 5E, circ_0021350, but not linear SOX6, was enriched, and the enrichment of miR-1207-3p in the circ_0021350 pulldown fraction was significantly higher than that of the NC group. In addition, the levels of miR-1207-3p in the glioma cells were notably increased after circ_0021350 silencing (Fig. 5F). Our findings indicated that circ_0021350 binds to miR-1207-3p and acts as a sponge in the glioma cells.

### Mir-1207-3p directly targets the oncogene PIK3R3

**Fig. 6:**
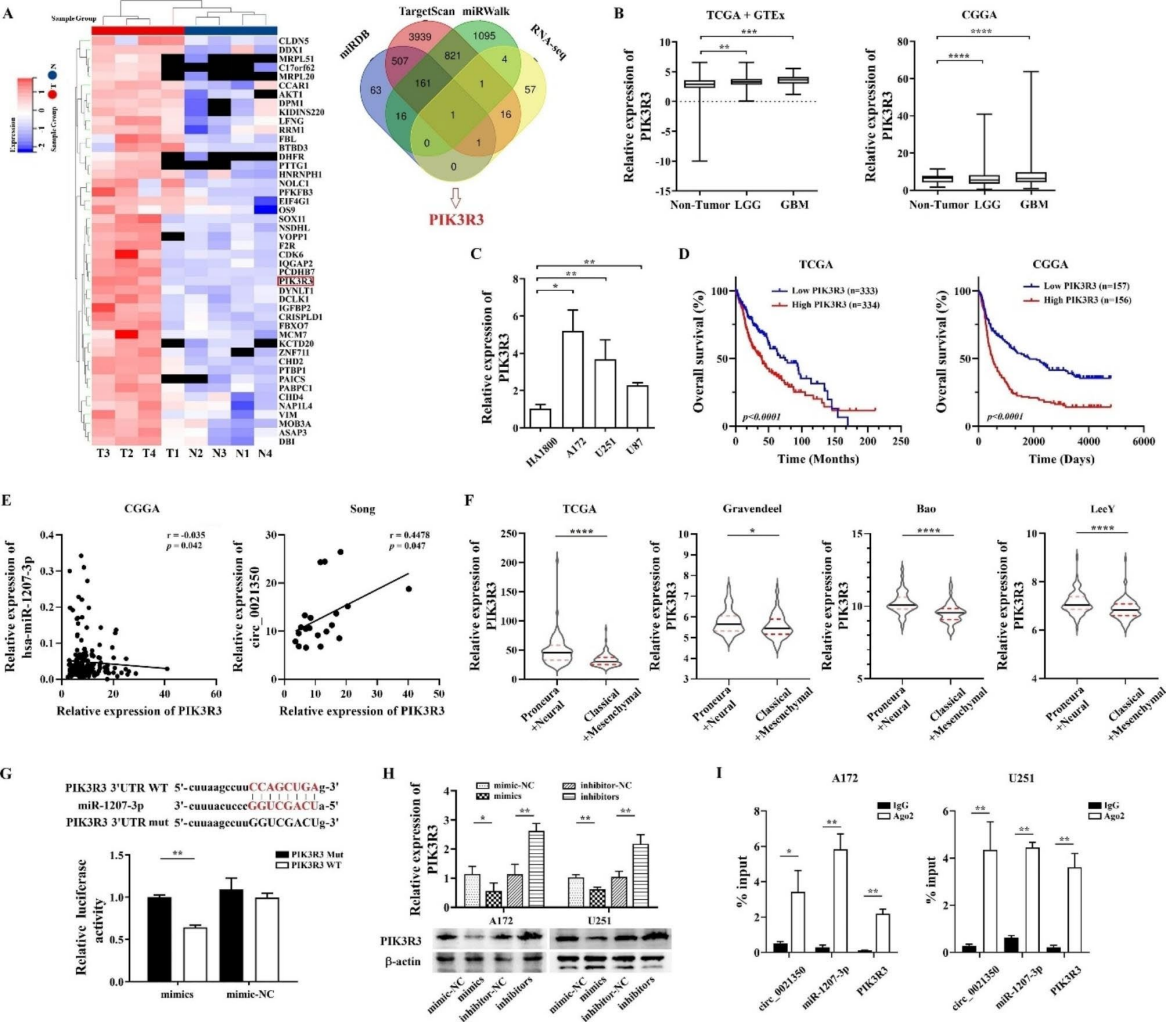
PIK3R3 is a target gene of miR-1207-3p. (**A**) screen of potential target genes of miR-1207-3p by bioinformatics tools and RNA-seq results. (**B**) The expression level of PIK3R3 in non-tumor and glioma tissues using TCGA and CGGA datasets. (**C**) Compare the expression of PIK3R3 in glioma cell lines and human astrocyte cells. (**D**) The prognostic potential of PIK3R3 in GBM by Kaplan-Meier analysis. (**E**) The correlation between miR-1207-3p/circ_0021350 and PIK3R3 in GBM was analyzed by Pearson’s correlation coefficients. (**F**) The relationship between PIK3R3 expression and GBM molecular subtypes was analyzed using online tool (GlioVis, http://gliovis.bioinfo.cnio.es/). (**G**) The binding between miR-1207-3p and PIK3R3 was verified by luciferase reporter assay. (**H**) qRT-PCR and western blot were used to detect the relevance of miR-1207-3p and PIK3R3 expression in glioma cells. Here, we used cropped blot images and the original images were in additional files. (**I**) RIP assay was performed to analysis the enrichment of circ_0021350, miR-1207-3p, and PIK3R3. ^*^P < 0.05, ^**^P < 0.01, ^***^P < 0.001, ^****^P < 0.0001

To select potential target genes of miR-1207-3p, we first screened mRNAs with MREs that could bind to miR-1207-3p in the TargetScan, miWalk, and miRDB databases and then intersected the candidates with mRNAs upregulated in the GBM tissues based on the RNA-seq analysis results (Fig. 6A, logFC > 2). PIK3R3 was identified as a target of miR-1207-3p. We compared the expression of PIK3R3 in LGG, GBM, and normal tissues based on TCGA and CGGA datasets and found that PIK3R3 expression levels were markedly higher in LGG and GBM than in normal tissues, especially in GBM (Fig. 6B). We also conducted qRT-PCR to compare the expression of PIK3R3 in the glioma cell lines and normal human astrocytes and discovered that PIK3R3 was simultaneously increased in the A172, U251, and U87 cells (Fig. 6C). When analyzing the prognostic value of PIK3R3 in GBM using the data from TCGA and CGGA databases, we found that high PIK3R3 expression was significantly associated with a poorer survival probability in gliomas (*P* < 0.0001; Fig. 6D). Pearson’s correlation analysis revealed that PIK3R3 and miR-1207-3p were negatively correlated, whereas PIK3R3 and circ_0021350 were positively correlated in GBM (Fig. 6E). Furthermore, PIK3R3 appeared to be preferentially expressed in molecular subtypes with better survival rates among patients with GBM. As shown in Fig. 6F, PIK3R3 levels were higher in proneural and neural subtypes than in mesenchymal and classical subtypes. Therefore, PIK3R3 is closely associated with glioma progression and can be used as a molecular subtype marker of GBM.

Next, we conducted a dual-luciferase reporter assay to confirm binding between PIK3R3 and miR-1207-3p. The results showed that co-transfection of the PIK3R3 3’UTR wt plasmid and miR-1207-3p mimics significantly reduced relative luciferase activity (Fig. 6G). Additionally, miR-1207-3p mimics significantly decreased the mRNA and protein expression of PIK3R3, whereas inhibitors significantly increased its expression (Fig. 6H). We further performed RIP assay, and the results showed enrichment of circ_0021350, miR-1207-3p, and PIK3R3 with the ant-Ago2 antibody (Fig. 6I). Collectively, these results demonstrated that PIK3R3 is a target gene of miR-1207-3p and is regulated by circ_0021350.

### Circ_0021350 facilitates the malignant phenotype of glioma cells by regulating PIK3R3

**Fig. 7:**
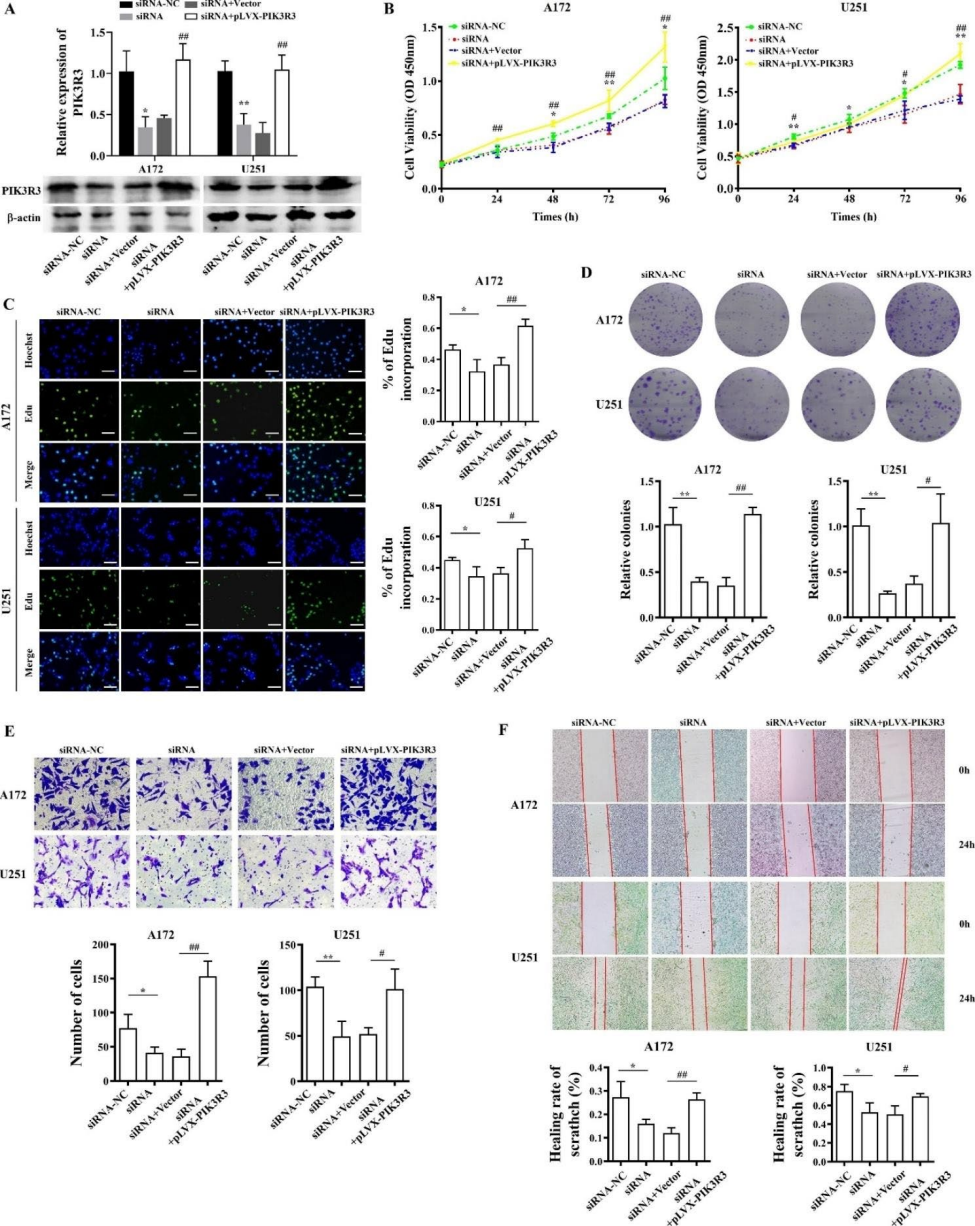
Circ_0021350 aggravated the malignant phenotype of glioma cells by regulating PIK3R3. (**A**) qRT-PCR was performed to measure PIK3R3 expression level in circ_0021350 knockdown cells and circ_0021350 knockdown with the PIK3R3 overexpressing vector cells. Here, we used cropped blot images and the original images were in additional files. (**B-C**)The proliferation rate of each group cells was examined by CCK-8 and EdU assays. Scale bars, 100 μm. (**D**) The clonogenic capacity of each group cells was examined by colony formation assay. (**E**) The invasion ability of each group cells was examined by transwell assay. (**F**) The migration ability of each group cells was examined by wound healing assay. ^*^P < 0.05, ^**^P < 0.01 siRNA-NC compared to the siRNA group; ^#^*P* < 0.05, ^##^*P* < 0.01 siRNA with empty vector group compared to siRNA with pLVX-PIK3R3 group

To further examine the functional interaction between circ_0021350 and PIK3R3, rescue experiments were performed by transfecting circ_0021350 knockdown cells with a PIK3R3 overexpressing vector (Fig. 7A). CCK-8, EdU, and colony formation assays indicated that PIK3R3 overexpression inhibited the decrease in cell proliferation and colony formation capacity induced by circ_0021350 knockdown (Fig. 7B-D). Transwell and wound-healing assays confirmed that PIK3R3 overexpression reversed the reduction in cell migration induced by circ_0021350 knockdown (Fig. 7E, F). These data suggested that PIK3R3 is positively regulated by circ_0021350 and that its overexpression promotes the malignant phenotype of glioma cells.

### Downregulation of circ_0021350 suppresses the growth of glioma cells in vivo

**Fig. 8:**
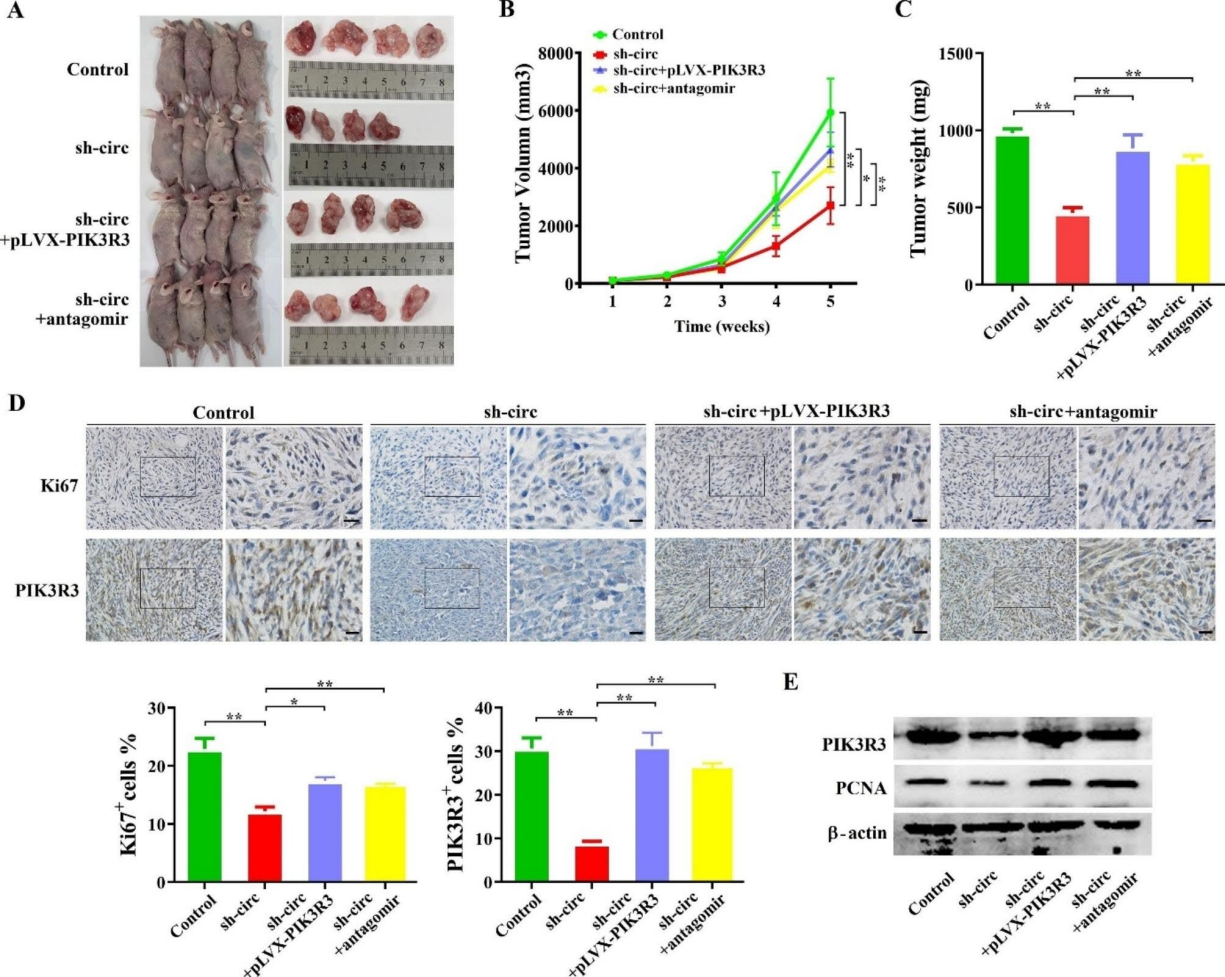
Circ_0021350 knockdown inhibited the growth of glioma cells in vivo. (**A**) Xenograft nude mice tumors in each group. Growth curve (**B**) and tumor weight (**C**) of each group. (**D**) The expression of Ki67 and PIK3R3 in xenograft tumors were detected by IHC staining. Scale bars, 50 μm. (**E**) The expression of of PCNA and PIK3R3 in xenograft tumors were detected by western blot analysis. Here, we used cropped blot images and the original images were in additional files. ^*^P < 0.05, ^**^P < 0.01

To explore the effects of circ_0021350 on glioma cell growth in vivo, the U251 cells were separated into four groups: blank control, Lv-circ_0021350-shRNAs transfected group, Lv-circ_0021350-shRNAs + miR-1207-3p antagomiR-co-transfected, and Lv-circ_0021350-shRNAs + pLVX-PIK3R3 co-transfected. The cells were subcutaneously injected into BALB/c nude mice (Fig. 8A). The xenograft models showed that circ_0021350 knockdown reduced tumor volume and weight, and this effect was inhibited by miR-1207-3p downregulation and PIK3R3 overexpression (Fig. 8B, C). Moreover, the IHC assay indicated that the downregulation of circ_0021350 reduced PIK3R3 expression. Ki67 expression was also found to be downregulated in the circ_0021350 knockdown group, and this trend was partially rescued by PIK3R3 overexpression or miR-1207-3p silencing (Fig. 8D). Western blot results showed that PIK3R3 protein levels were reduced by circ_0021350 knockdown, whereas PIK3R3 overexpression or miR-1207-3p silencing restored them, which is consistent with the IHC results. Additionally, the expression of PCNA in the circ_0021350 knockdown group was much lower compared to the other three groups, indicating that circ_0021350 knockdown inhibited cell proliferation was also restored by PIK3R3 overexpression or miR-1207-3p silencing (Fig. 8E).

## Discussion

According to the theory of Hanahan [19], there are ten key biological capabilities that are acquired during the multistep development of tumors. Abnormal expression of circRNAs in the glioma cells has been reported to participate in all these steps, including the maintenance of proliferation signals, avoidance of growth suppressors and cell death, promotion of metastasis, induction of angiogenesis and inflammation, and disordered cellular energy [14, 20–23]. Therefore, identifying more effective circRNAs will provide ideas for targeted therapy of GBM. In this study, we suggested that a novel circRNA, circ_0021350, which has never been discussed to the best of our knowledge, is highly expressed in GBM. Cell function experiments verified that circ_0021350 promoted the proliferation, colony formation, and metastatic abilities of the glioma cells. These findings highlighted circ_0021350 as a potential therapeutic target and feasible biomarker for patients with GBM.

Circ_0021350 is generated by the reverse splicing of exons 10 and 11 of SOX6. Sanger sequencing, RT-PCR, and RNase R treatment confirmed the presence of back-splicing sites and circular structures. FISH results showed that circ_0021350 was located in both the cytoplasm and nucleus. Most circRNAs located in the cytoplasm contain miRNA response elements that perform biological functions by binding miRNAs and regulating their target genes [24]. Accordingly, miRNA targeting analysis was performed, and miR-1207-3p was identified as a downstream miRNA of circ_0021350. MiR-1207-3p was recently shown to hinder prostate cancer cell proliferation and metastasis [25]; however, its role in GBM remains unknown. Our data indicated that miR-1207-3p is underexpressed in GBM and is sponged by circ_0021350 to further regulate the malignant phenotypes of GBM. These findings highlighted miR-1207-3p as a tumor suppressor that interacts with circRNAs. In contrast, circ_0021350, also located in the nucleus, suggests probable binding between circ_0021350 and DNA- and RNA-binding proteins, similar to the relationship between circFAT1 (e2) and the YBX1 protein [26]. Therefore, the function of circ_0021350 in the nucleus should be investigated in future studies.

We also revealed that PIK3R3 is a downstream target of miR-1207-3p. PIK3R3 is a regulatory subunit of PI3K that is involved in cellular genesis, proliferation, differentiation, apoptosis, and metabolism. Recently, PIK3R3 was found to be upregulated in liver cancer; it activates Akt signaling to control cancer growth by regulating CDKN1C and SMC1A [27]. A previous study revealed that PIK3R3 promotes chemotherapeutic sensitivity of colorectal cancer via the NF-κB/TP pathway [28]. Available data have also shown aberrant expression of PIK3R3 in ovarian cancer and sarcoma, indicating a promoting role of PIK3R3 in oncogenesis [29, 30]. Consistent with these reports, our study also demonstrated the high expression of PIK3R3 in GBM and its potential for predicting poor prognosis. In this study, we found that PIK3R3 expression was negatively correlated with miR-1207-3p expression, whereas it was positively correlated with circ_0021350 expression in the tissues from patients with GBM. Moreover, RIP analysis indicated enrichment of circ_0021350, miR-1207-3p, and PIK3R3 in the glioma cells. Overexpression of PIK3R3 rescues the glioma cell phenotype upon circ_0021350 knockdown both in vitro and in vivo. These findings highlighted the existence of a circ_0021350/miR-1207-3p/PIK3R3 axis and confirmed that the oncogenic role of circ_0021350 in GBM occurs, at least partly, through the regulation of PIK3R3.

**Fig. 9:**
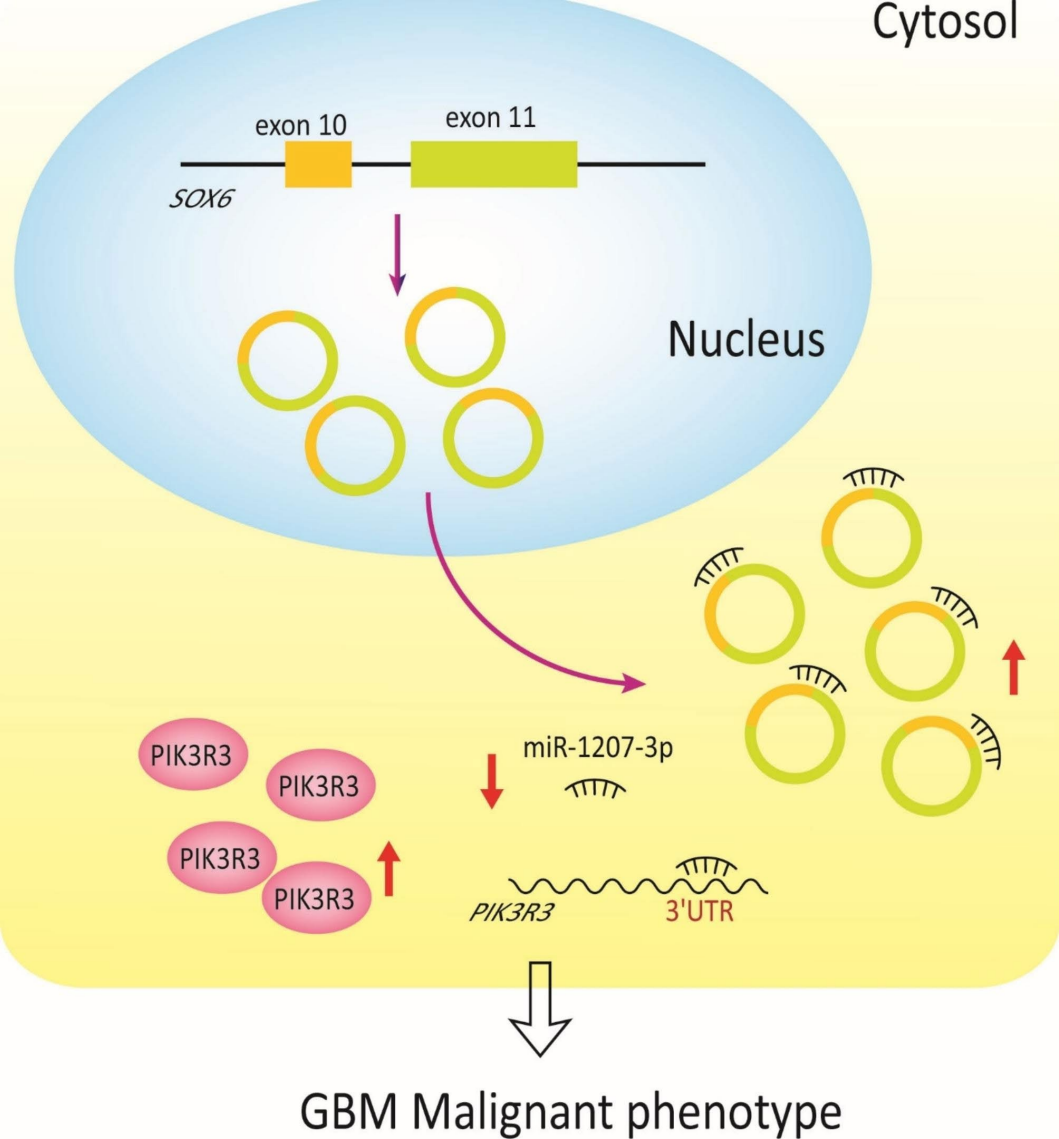
Illustration of the mechanism of circ_0021350 on promoting GBM malignant phenotype regulating miR-1207-3p/PIK3R3

Taken together, our findings provided strong evidence that circ_0021350 acts as a potential biomarker that affects the biological behavior of the GBM cells. It maintains the oncogenic functions of PIK3R3 by sponging miR-1207-3p (Fig. 9), thereby promoting aggressive tumor biology in cells and aggravating GBM formation and development. Thus, the suppression of circ_0021350 may help improve the prognosis of patients with GBM and may be a novel target for the clinical treatment of GBM in the future. This study provided good application prospects for the molecular therapy of GBM and laid a new foundation for an in-depth understanding of tumor mechanisms.

However, this study had some limitations. First, our clinical sample size was relatively small, and there may have been some bias. A larger patient cohort should be used for further verification and exploration of the correlation between circ_0021350 and the clinical characteristics of patients with GBM. Second, the cell lines do not fully reflect the genotypes and phenotypes of the primary tumors. Therefore, primary patient-derived GBM cells should be used for phenotypic validation. This will be the focus of our future research.

### Electronic supplementary material

Below is the link to the electronic supplementary material.

Supplementary Material 1

## Data Availability

The datasets used and/or analyzed during the current study are available from the corresponding author upon reasonable request. The datasets generated from The TCGA database (http://tcga-data.nci.nih.gov/tcga), CGGA database (http://www.cgga.org.cn), and GEO database (GSE145510, https://www.ncbi.nlm.nih.gov/geo/) analyzed during the current study are publicly available.

## Abbreviations

GBM: Glioblastoma
circRNAs: Circular RNAs
miRNAs: MicroRNAs
ncRNAs: Non-coding RNAs
FISH: Fluorescence in situ hybridization assay
qRT-PCR: Quantitative real-time polymerase chain reaction
CCK-8: Cell counting kit 8
EdU: 5-ethynyl-29-deoxyuridine
RIP: RNA binding protein immunoprecipitation
PIK3R3: Phosphoinositide-3-kinase regulatory subunit 3
