# Supplementary material for: Circ_0021350 plays an oncogene role by regulating miR-1207-3p/PIK3R3 in glioblastoma
Source: BMC Cancer. 2023 Aug 29;23:808. doi: 10.1186/s12885-023-11263-w (PMC10463850; doi:10.1186/s12885-023-11263-w)
Supplement: Supplementary file 1 — Supplementary Material 1 [file 12885_2023_11263_MOESM1_ESM.pdf]

Circ_0021350 plays an oncogene role by regulating miR-1207-3p/ PIK3R3 in glioblastoma

Cheng Tan[1], Jun Wei[2], Zhaohui Li[3], Nan Tian[4], Zhengming Wang[3], Guan Wang[3], Liang Han[5*], Yu Tian[3*]

TableS 1. The sequence of designed primers

Type	Primer name	Sequences (5′-3′)
Divergent primer	circ_0021350-Div-F	AACAACGGCAGCAAATGGAC
	circ_0021350-Div-R	GCTGCTCCCGTAAACTGATC
Convergent primer	circ_0021350-Con-F	GAACAGAAAAAACTGGCAGC
	circ_0021350-Con-R	GAAGTTGCTGCTGTTGTCTC
qRT-PCR primers	β-actin-F	CTCCATCCTGGCCTCGCTGT
	β-actin-R	GCTGTCACCTTCACCGTTCC
	PIK3R3-F	TACAATACGGTGTGGAGTATGGA
	PIK3R3-R	TCATTGGCTTAGGTGGCTTTG
	U6-F	CGCAAGGATGACACGCAA
	U6-R	GTGCAGGGTCCGAGGT
	miR-1207-3p Reverse transcription	GTCGTATCCAGTGCAGGGTCCGAGGTATTC GCACTGGATACGACGAAATG
	miR-1207-3p-F	GCGTCAGCTGGCCCT
	miR-1207-3p-R	CAGTGCAGGGTCCGAGGT
	Forward primer	CGCCGTGAGGTAGTAGTTTGT
RNA pull down probe	circ_0021350-5' bio	TCAGGTGTACCTGGATCTGT
Fish probe	circ_0021350-5' cy3	TCTCAGGTGTACCTGGTTTGTATG

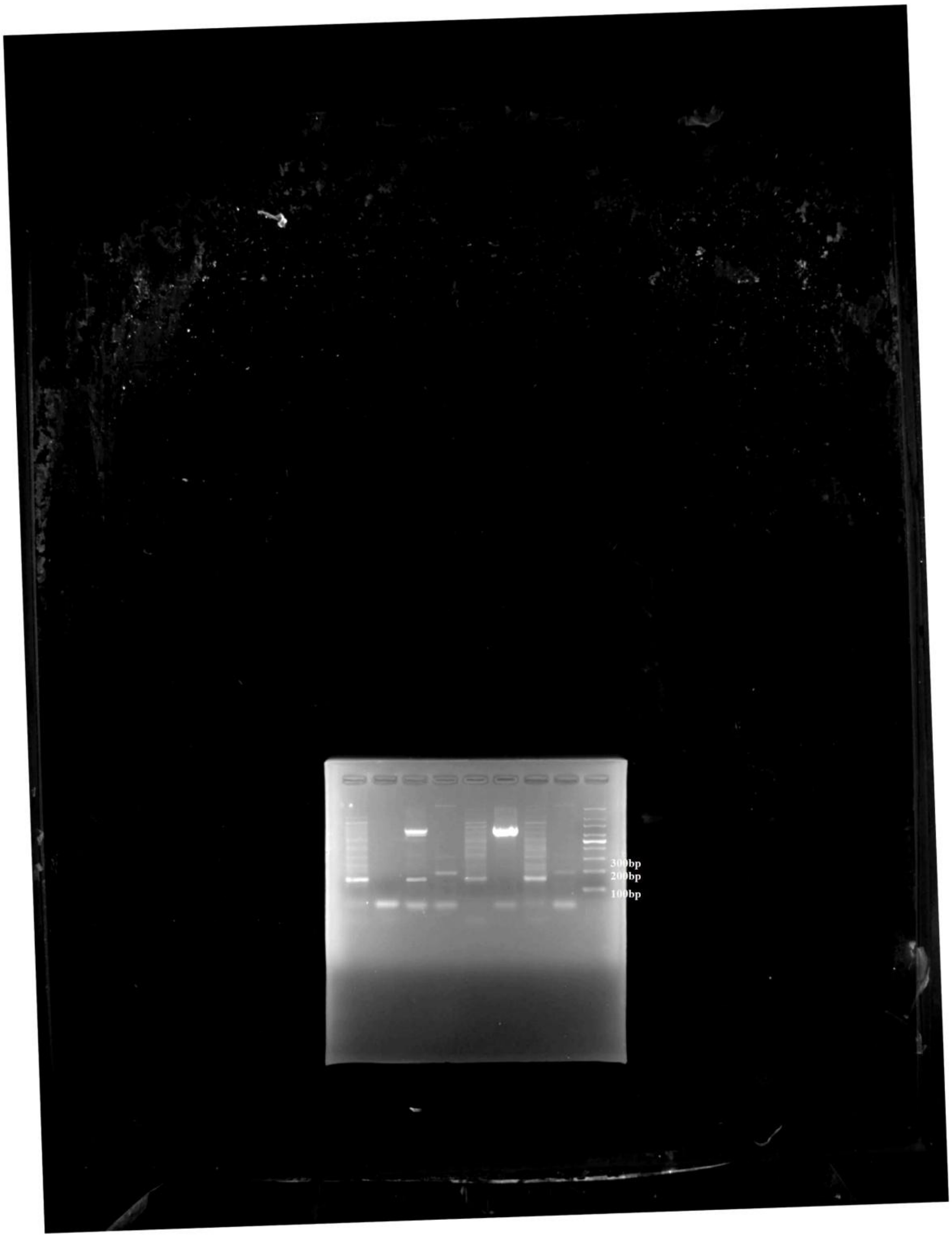

Full-lengh gel of Figure 2D

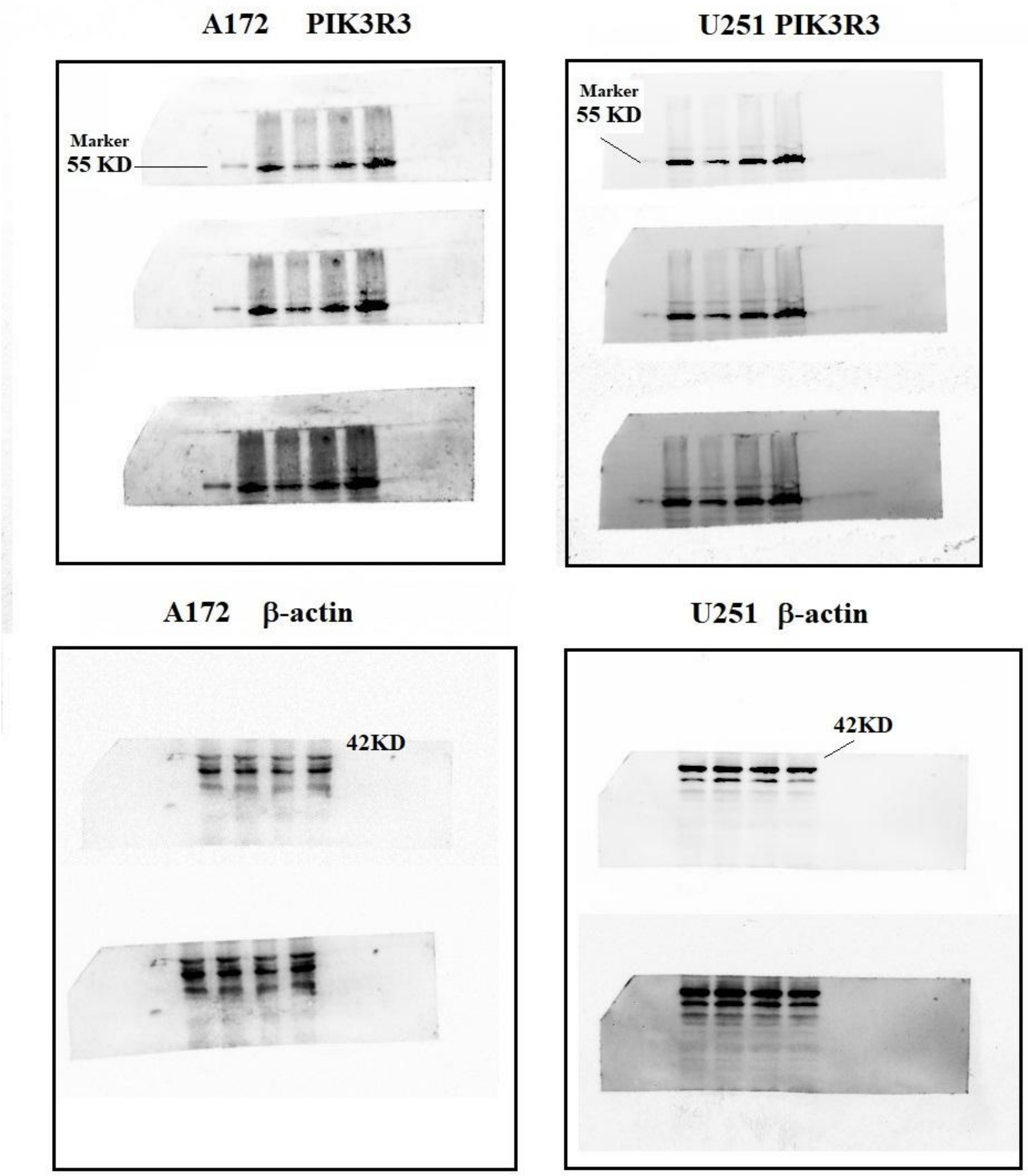
A172 PIK3R3
Marker
55 KD
U251 PIK3R3
Marker
55 KD
A172 β-actin
42KD
U251 β-actin
42KD

Figure 6H

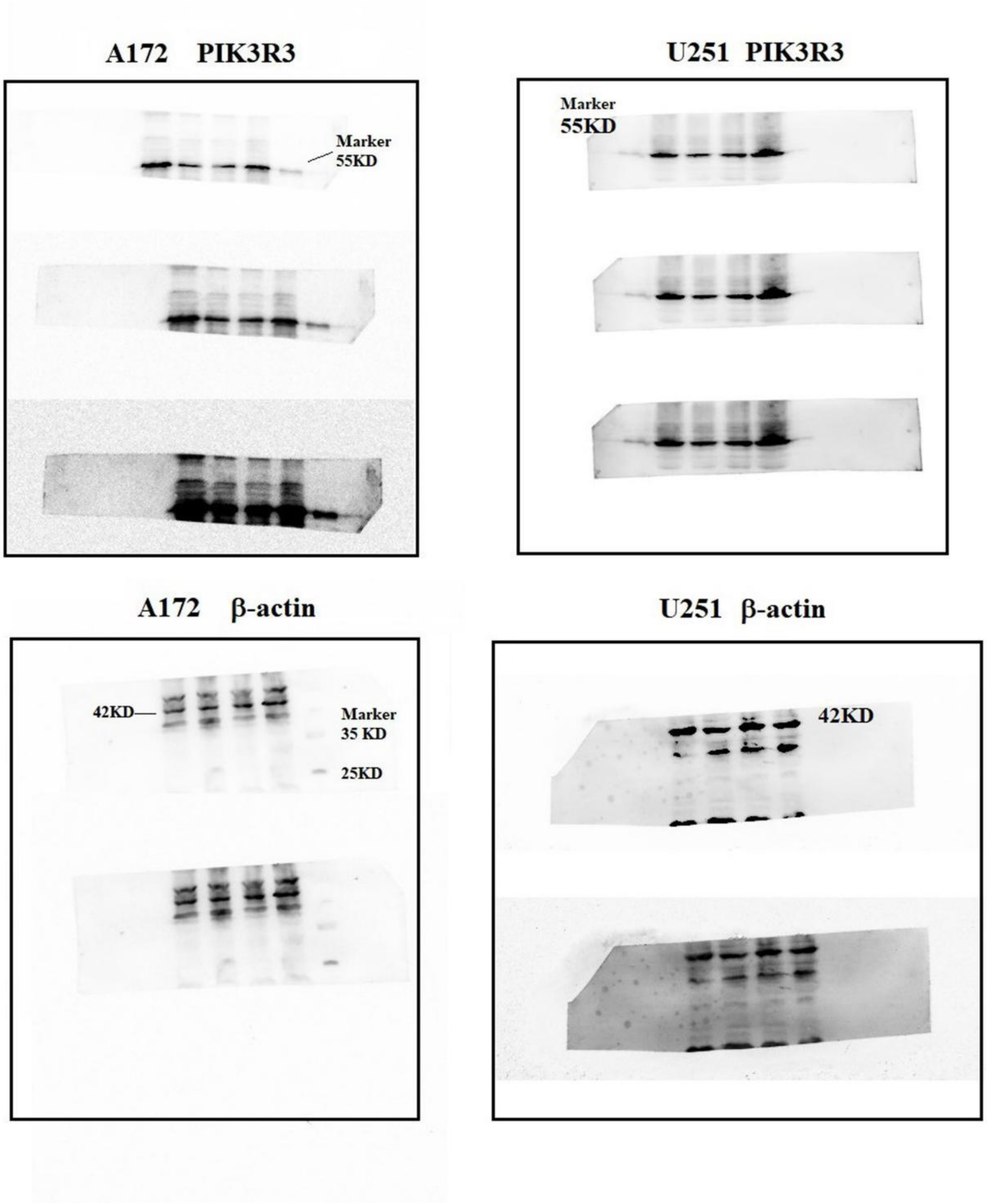
A172 PIK3R3
Marker
55KD
U251 PIK3R3
Marker
55KD
A172 β-actin
42KD
Marker
35 KD
25KD
U251 β-actin
42KD

Figure 7A

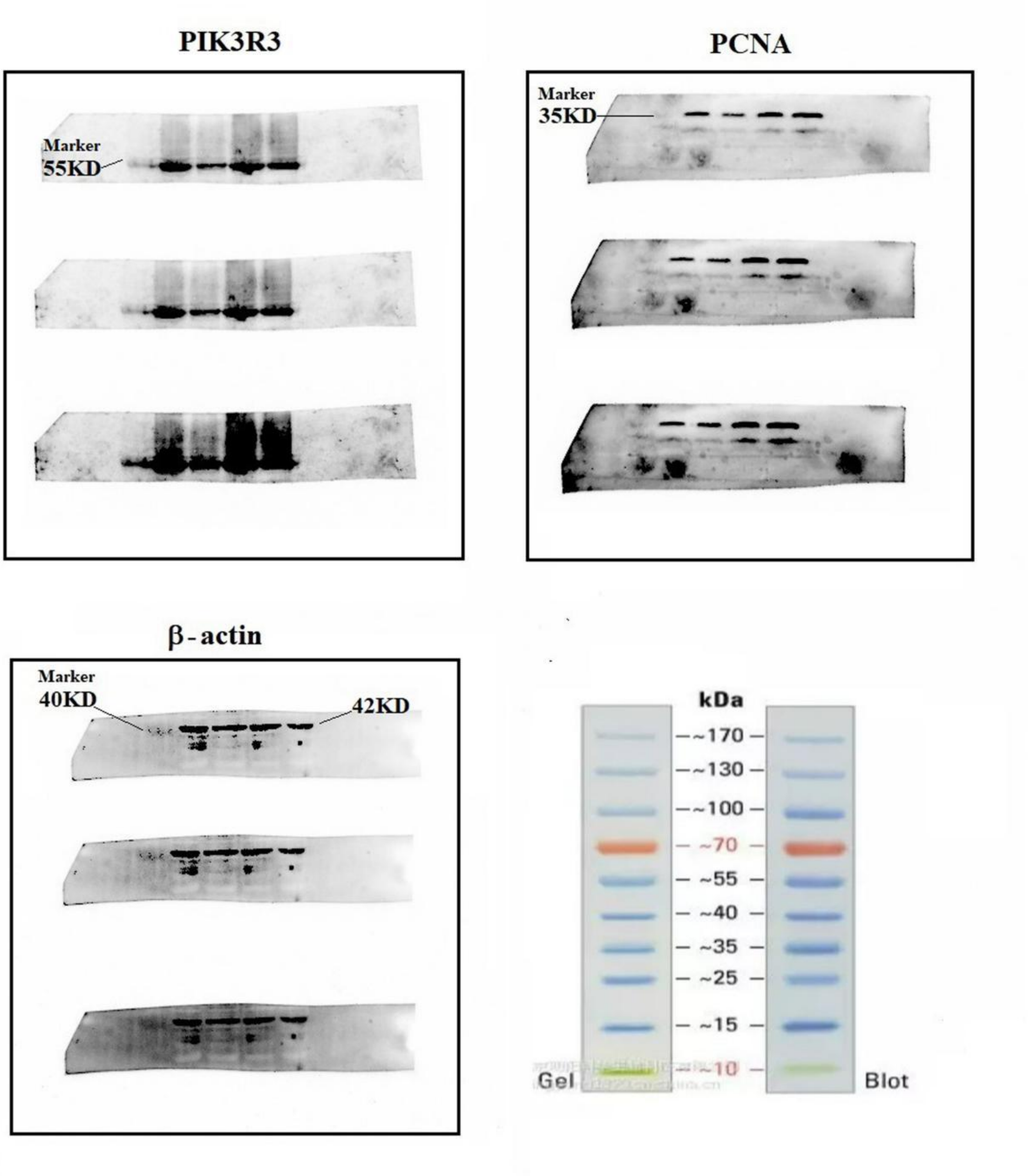
PIK3R3
Marker
55KD
PCNA
Marker
35KD
β-actin
Marker
40KD
42KD
kDa
~170
~130
~100
~70
~55
~40
~35
~25
~15
~10
Gel
Blot

Figure 8E

PIK3R3

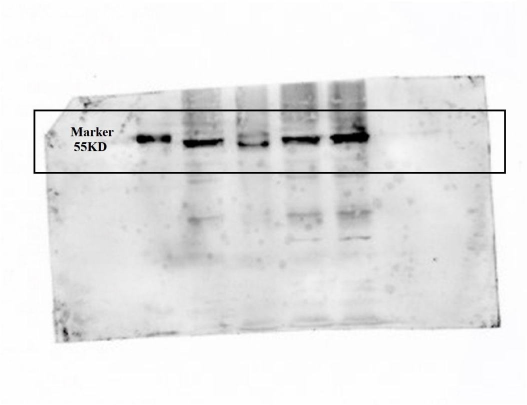

PCNA

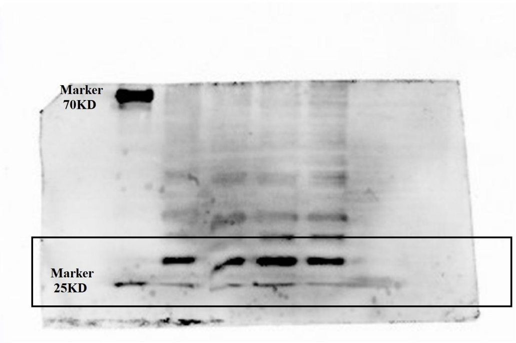

β-actin

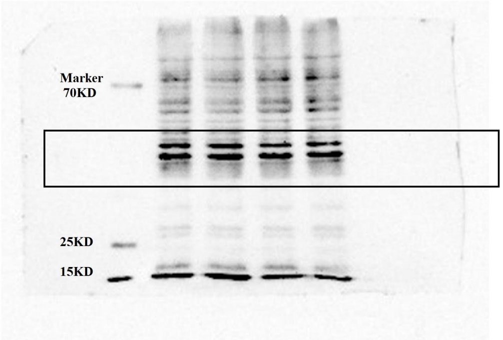

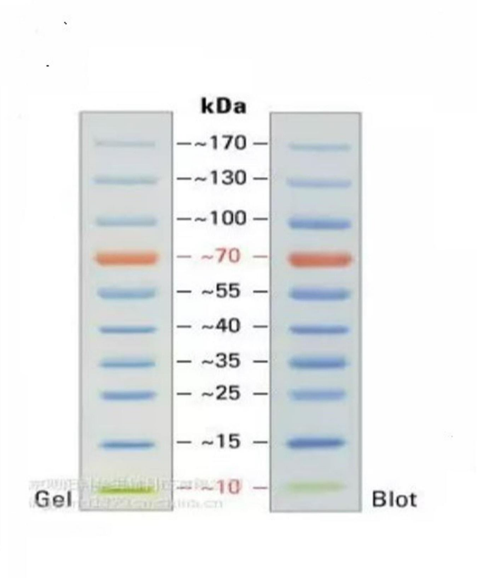

Unprocessed blot for each antibody to confirm specific detection
